# Supplementary material for: Routine childhood immunization coverage, timeliness, dropout, and missed opportunities in Northern Ghana: Evidence from a community-based survey in Tamale Metropolis, Ghana
Source: PLOS Glob Public Health. 2026 Apr 10;6(4):e0005806. doi: 10.1371/journal.pgph.0005806 (PMC13068253; doi:10.1371/journal.pgph.0005806)
Supplement: S1 File — (DOCX) [file pgph.0005806.s002.docx]

DATA COLLECTION TOOL FOR ROUTINE IMMUNIZATION COVERAGE SURVEY IN TAMALE, GHANA

| SECTION A: BACKGROUND | | | | |
| --- | --- | --- | --- | --- |
| S/N | QUESTIONS | FILTERS | RESPONSE | SKIP |
|  | Address |  |  |  |
|  | Nearest health facility | Name |  |  |
|  | Age of child (completed months) | Months | 99: Don’t know |  |
|  | Sex of child |  | 1. Male 2. Female |  |
|  | Place attended ANC |  | 88: None |  |
|  | Place of delivery |  | 1. Public facility 2. Private facility 3. Home/ TBA   99. Don’t know | Question 7  Question 7 |
|  | Name of facility |  | __________________________________ |  |
|  | Relationship of respondent to child |  | 1. Mother 2. Father 3. Other (specify) |  |
|  | Sex of caregiver/respondent |  | 1. Male 2. Female |  |
|  | Educational level of respondent |  | 1. Primary 2. Secondary 3. Tertiary 4. Other (specify) |  |
|  | Occupation of respondent |  | 1. Employed 2. Unemployed |  |
|  | Age of Respondent | Years | 99: Don’t know |  |
|  | Home-based or vaccination card record |  |  |  |
|  | Has vaccination card |  | 1. Yes 2. No   99. Don’t know | QUESTION 16  QUESTION 15  QUESTION 15 |
|  | Why respondent does not have a vaccination card |  | 1. Lost 2. Destroyed 3. never vaccinated 4. Other (specify) | QUESTION 55 (Any response) |
|  | Is card the original one received or replacement/copy |  | 1. Original 2. Replacement   99. Don’t know | QUESTION 19 |
|  | Did you pay for replacement/copy |  | 1. Yes 2. No   99. Don’t know |  |
|  | Date of birth (as recorded on card) |  | DD/MM/YY |  |
| SECTION B: VACCINATION COVERAGE USING CARD | | | | |
|  | Penta 1 |  | 1. Yes 2. No | QUESTION 21 |
|  | Penta 1 | Date | DD/MM/YY |  |
|  | PCV1 |  | 1. Yes 2. No | QUESTION 23 |
|  | PCV 1 | Date | DD/MM/YY |  |
|  | OPV1 |  | 1. Yes 2. No | QUESTION 25 |
|  | OPV1 | Date | DD/MM/YY |  |
|  | Rota 1 |  | 1. Yes 2. No | QUESTION 27 |
|  | Rota 1 | Date | DD/MM/YY |  |
|  | Penta 2 |  | 1. Yes 2. No | QUESTION 29 |
|  | Penta 2 | Date | DD/MM/YY |  |
|  | PCV 2 |  | 1. Yes 2. No | QUESTION 31 |
|  | PCV 2 | Date | DD/MM/YY |  |
|  | Polio 2 |  | 1. Yes 2. No | QUESTION 33 |
|  | Polio 2 | Date | DD/MM/YY |  |
|  | Rota 2 |  | 1. Yes 2. No | QUESTION 35 |
|  | Rota 2 | Date | DD/MM/YY |  |
|  | Penta 3 |  | 1. Yes 2. No | QUESTION 37 |
|  | Penta 3 | Date | DD/MM/YY |  |
|  | PCV 3 |  | 1. Yes 2. No | QUESTION 39 |
|  | PCV 3 | Date | DD/MM/YY |  |
|  | Polio 3 |  | 1. Yes 2. No | QUESTION 41 |
|  | Polio 3 | Date | DD/MM/YY |  |
|  | Rota 3 |  | 1. Yes 2. No | QUESTION 43 |
|  | Rota 3 | Date | DD/MM/YY |  |
|  | MR 1 |  | 1. Yes 2. No | QUESTION 45 |
|  | MR 1 | Date | DD/MM/YY |  |
|  | MR 2 |  | 1. Yes 2. No | QUESTION 47 |
|  | MR 2 | Date | DD/MM/YY |  |
|  | YF |  | 1. Yes 2. No | QUESTION 49 |
|  | YF | Date | DD/MM/YY |  |
|  | IPV |  | 1. Yes 2. No | QUESTION 51 |
|  | IPV | Date | DD/MM/YY |  |
|  | Men A |  | 1. Yes 2. No | QUESTION 53 |
|  | Men A | Date | DD/MM/YY |  |
| SECTION C: CAREGIVER RECALL OF VACCINATION | | | | |
|  | Has child ever received any vaccinations, drops in the mouth or injections in the arm |  | 1. Yes 2. No |  |
|  | Has the child ever received any “vaccination drops in the mouth” – that is, polio? |  | 1. Yes 2. No   99. Don’t know | QUESTION 56  QUESTION 56 |
|  | How many times did child receive the polio (drops) |  | ______________ |  |
|  | Has the child ever received an injection on the upper outer thigh? – that is a penta |  | 1. Yes 2. No   99. Don’t know | QUESTION 58  QUESTION 58 |
|  | How many times |  | ___________ |  |
|  | Has the child ever received PCV vaccine? |  | 1. Yes 2. No   99. Don’t know | QUESTION 60  QUESTION 60 |
|  | How many times? |  | _____________ |  |
|  | Has the child ever received Rotavirus vaccine? |  | 1. Yes 2. No   99. Don’t know | QUESTION 62  QUESTION 62 |
|  | How many times |  | __________ |  |
|  | Do you think child has received all the recommended vaccines for their age? (Data collector to determine) |  | 1. Yes 2. No   99. Don’t know | QUESTION 63 |
|  | Why hasn't the child had all recommended vaccines? (Record all that apply, don't read out options, listen carefully to caregiver) | 1. Vaccination center too far 2. Hidden costs (pay for card, charging for vaccination) 3. Cost of transport 4. Fear of missing work 5. Time of immunization inconvenient, what do they mean? 6. Long wait 7. Fear of side effects 8. Unaware of the need for immunization 9. Unaware of need to return for subsequent doses 10. Mother too busy 11. Family problem including illness of mother 12. Rumors/myths about vaccines 13. No faith in immunization 14. Child not living with mother 15. Place of immunization unknown 16. time of immunization unknown 17. time of immunization inconvenient 18. Vaccinator absent 19. Inadequate information on immunization 20. Vaccine stock-outs/not available 21. Fear of covid 22. Thought was complete 23. Other (Specify) | |  |
| 50 | Where did child receive most recent vaccinations? | 1. Static point in health facility 2. Outreach point in neighborhood 3. Other (specify) | | QUESTION 51 |
| 51 | Name of static point where child received most recent vaccines | ___________________________________________________________ | |  |
