# Supplementary material for: Routine childhood immunization coverage, timeliness, dropout, and missed opportunities in Northern Ghana: Evidence from a community-based survey in Tamale Metropolis, Ghana
Source: PLOS Glob Public Health. 2026 Apr 10;6(4):e0005806. doi: 10.1371/journal.pgph.0005806 (PMC13068253; doi:10.1371/journal.pgph.0005806)
Supplement: Attachment — Submitted filename: REVISED RESPONSE_TO_REVIEWER_COMMENTS__24_02.pdf [file pgph.0005806.s005.pdf]

School of Population and Global Health

The University of Western Australia

Perth, Australia

24 February 2026.

Dr. Catherine Kyobutungi

The Editor-in-Chief
PLOS Global Public Health

Dear Dr., Kyobutungi,

Response to re viewers:Manuscript: PGPH-D-25R1-02884— Routine Childhood Immunization Coverage, Timeliness, Dropout, and Missed Opportunities in Northern Ghana: Evidence from a Community-Based Survey in Tamale Metropolis, Ghana.

We thank you and the reviewers for your careful evaluation of our manuscript and for the constructive feedback provided. We have revised our manuscript accordingly and provide responses to all comments below. Changes made in the manuscript are highlighted in the tracked version.

All the editorial requests have been addressed.

We thank the reviewers and editor again for their valuable feedback and patience during the review process. We believe the revisions have addressed all major and minor comments and have improved the clarity, rigor, and utility of our manuscript.

Yours Sincerely,

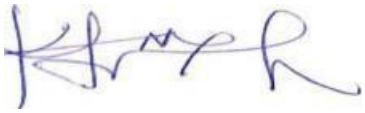

Matthew Konlan (Corresponding Author)

REVIEWER'S COMMENTS	SECTION	RESPONSE TO REVIEWERS' COMMENTS
**Reviewer #1**		
Line 1: Capitalize the "t" in "Timeliness"	Title	Thank you for the correction, the title has however been revised based on a suggestion by another reviewer and now reads as: "Routine Childhood Immunization Coverage, Timeliness, Dropout, and Missed Opportunities in Northern Ghana: Evidence from a Community-Based Survey in Tamale Metropolis, Ghana."<br>.
Line 52 Place "(MoV)" after "Missed opportunities for vaccinations..." This allows you to then use the abbreviation, "MoV" as an abbreviation later in the text.	Abstract	Thank you for the helpful suggestion. We have now inserted "(MoV)" immediately after the first mention of missed opportunities for vaccination in Line 48.
Line 57 Change to "...Penta1 to Penta3."		Thank you for the suggestion. We reanalysed the data and have revised the text accordingly and changed the phrase to **"…**Penta1 to Penta3." as recommended.
**Reviewer 2**		.
Analytical depth and statistical rigour<br>- The analysis is appropriate for a descriptive cross-sectional study. However, it would be strengthened by including basic inferential analyses (e.g., bivariate or multivariable logistic regression) to explore factors associated with dropout or missed opportunities for vaccination.<br>If inferential analysis is not feasible, please explicitly justify this choice		Thank you for the suggestion. Our study was designed as a descriptive, diagnostic assessment to inform the design of solutions at the subdistrict and CHPS level. We, therefore, elected not to conduct inferential modelling. We have clarified this rationale in the Methods and Limitations and emphasised that our intent was to describe patterns and pinpoint operational bottlenecks, consistent with STROBE guidance for cross-sectional studies. In the study design and population section, we have specifically

and emphasise that the study's intent is descriptive and diagnostic.		indicated that "We conducted a descriptive cross-sectional study of 1,020 children aged 12-59 months to enable us to estimate full immunization coverage, describe immunization patterns, and pinpoint operational bottlenecks in routine childhood immunization in the Tamale Metropolis from November to December 2022." We acknowledge the limitation of this study design in our study strength and limitations as well.
Clarify whether survey weights and cluster design effects were accounted for in the statistical analysis. This is particularly relevant given the multi-stage sampling design.<br>- The manuscript does not report any sensitivity or robustness checks, which would strengthen confidence in the estimates given the reliance on vaccination cards and potential missing data.		Thank you for raising this important point. We have now clarified how survey weights and the multi-stage sampling structure were handled in the analysis. Specifically, we applied sampling weights for all analyses using the full dataset. However, for sub-analyses restricted to only a portion of the sample, weights were not applied because the resulting subset was no longer representative, and weighting would have produced biased or unstable estimates. These clarifications and additional details have been incorporated in the Statistical Analysis section as follows: "In our analysis and reporting of the results, we weighted the outcomes involving all respondents. Given the potential for weighting to under- or over-estimate coverage in sub-analyses because the sample is no longer representative of the population, we did not weight samples when the unit of analysis was a subset of the total sample. Denominators vary by vaccine, as coverage was calculated based on the number of children old enough to

		be eligible for that specific dose at the time of the survey.”
The current Data Availability Statement (“available upon reasonable request”) does not fully meet PLOS’s open data policy. Please deposit the anonymised dataset in a public repository or provide a valid justification for restricted access (e.g., ethical or legal constraints).<br>- Update the statement accordingly with a persistent link or DOI.	Data availability and transparency	Thank you for this important clarification request. We have now updated the Data Availability Statement to meet PLOS’ open data requirements as follows: “All relevant data are within the manuscript and its supporting information files.” An anonymised version of the dataset has been deposited as a supporting information file labelled “S1 Data”in the submission
Ethical approval was obtained (Navrongo Health Research Centre IRB), informed consent was secured, and data availability is declared.<br>- Please include the date of ethical approval.<br>- Please explain why verbal consent rather than written consent was used.	Ethical and Reporting Standards	Thank you for this important comment. We have now updated the Ethical Considerations section to include the date of ethical approval from the Navrongo Health Research Centre IRB, which is 16th December 2022. We have also added a justification for the use of verbal rather than written informed consent: “Verbal informed consent was approved by the IRB and used in place of written consent because the study posed minimal risk, involved no collection of personal identifiers, and was conducted in settings where varying literacy levels could make written consent burdensome or inadvertently coercive.”
Expand the discussion to include policy and programmatic implications. For instance, how could your findings inform Ghana’s EPI performance reviews or IA2030 monitoring frameworks?<br>Reflect briefly on health system and social determinants that may underlie dropout and missed	Discussion and implications	Thank you for this valuable suggestion. We have now expanded the Discussion section to articulate the policy and programmatic implications of our findings, including how they can inform Ghana’s EPI performance reviews and align with the Immunization Agenda 2030 (IA2030) monitoring framework. Specifically, we indicate that: “This

opportunities (e.g., access barriers, service quality, gender dynamics).		study highlights strengths and gaps in routine childhood immunization delivery in Tamale that are relevant to Ghana's EPI performance reviews and IA2030 monitoring. High coverage for early vaccines suggests successful initial contact with immunization services. However, the marginal declines in coverage for later doses, high dropout rates and missed opportunities for vaccination, points to weaknesses in continuity of care. There is the need for EPI monitoring frameworks to place greater emphasis on second year-of-life vaccination, dropout tracking, timeliness, and MoV indicators, rather than focusing predominantly on early-dose coverage. Sub-district variation in performance further underscores the value of decentralized, data-driven reviews that enable targeted corrective actions in underperforming areas."
The conclusion somewhat restates findings rather than extrapolating actionable recommendations.	Conclusion	Thank you for this constructive comment. We have revised the *Conclusion* section to move beyond restating the findings and to provide clear, actionable programmatic recommendations. The updated conclusion now highlights the specific actions needed to strengthen routine immunisation in the Tamale Metropolis, namely improving tracking systems, enhancing provider capacity, and deepening community engagement to reduce dropout and missed opportunities for vaccination. It also situates these actions within the broader strategic framework of the

		Immunization Agenda 2030, emphasising how targeted efforts are required to restore and sustain high coverage, particularly for second-year-of-life vaccines. Our conclusion now reads as follows: "The study identifies significant challenges in vaccination coverage among children aged 12-59 months in the Tamale Metropolis, characterized by low coverage for vaccines given at 18 months and high dropout rates and MoVs, particularly for the rotavirus vaccine. Despite high initial vaccine uptake, sustaining consistent immunization schedules remains a challenge. These gaps highlight the need for improved tracking, healthcare provider training, and community engagement to ensure that all children receive the full benefits of immunization programs. Addressing these challenges and strengthening tracking systems and community engagement will be essential to achieve the targets of the Immunization Agenda 2030."
The limitations are clearly acknowledged, but you could strengthen this section by discussing potential biases (recall bias, card completeness) and the cross-sectional design's inability to infer causality.	Limitations section	Thank you for this helpful suggestion. We have strengthened the *Limitations* section by explicitly discussing potential sources of bias and the implications of the cross-sectional design. In the revised text, we now highlight the possibility of recall bias among caregivers without home-based records, the risk of misclassification due to incomplete vaccination cards, and the potential underestimation of coverage and missed opportunities for vaccination (MoV) in children lacking

		documentation. We also note that reliance on card data may limit representativeness and introduce measurement error. In addition, we explicitly acknowledge that, due to the cross-sectional design, causal relationships cannot be inferred.
**Minor Comments**		
Language and style:<br>1. - The manuscript is written in clear, standard English, but a light copyedit is recommended to correct minor grammatical, punctuation, and stylistic inconsistencies (e.g., “optimum” → “optimal,” consistent vaccine name formatting, and spacing before citations)	Entire document	Thank you for the comment. We have now conducted a light copyedit of the full manuscript to address minor grammatical, punctuation, and stylistic inconsistencies. Specifically, we corrected wording such as “optimum” to “optimal,” standardised vaccine name formatting throughout the text, ensured consistent spacing before citations, and harmonised terminology across sections
**Presentation:**<br>- Ensure consistent use of terms such as dropout rate, missed opportunities, and coverage across tables and text.<br><br>- Some sentences in the Discussion are repetitive and could be streamlined for conciseness.	Entire document	Thank you for this helpful observation. We have revised the manuscript to ensure consistent use of key terms, including dropout rate, missed opportunities for vaccination (MoV), and coverage, across all tables, figures, and text. Terminology has been standardised according to WHO definitions to avoid ambiguity.<br>We also undertook a light revision of the discussion to remove repetitive statements and streamline the narrative. Redundant phrases have been eliminated, and the section now presents a more concise and coherent interpretation of the findings, with clearer linkages to policy and programmatic implications.

- Ensure consistent formatting (some entries mix sentence-case and title-case).	References	Thank you for highlighting this formatting issue. We have now reviewed the entire manuscript, tables, headings, and figure labels to ensure consistent formatting, particularly with respect to the use of sentence case vs title case. Terminology, variable names, and section headings have been standardised for uniformity throughout the document.
Double-check URLs (e.g., WHO links) for functionality. 31,32 and 35<br><br>- Reference [37] author name should be capitalised ("Singh, J." not "singh, j.").<br>2.		Thank you for these helpful observations. We have now checked all URLs, including the WHO links referenced in [31], [32], and [35], to ensure they are functional, current, and correctly formatted. Any broken or outdated links have been updated accordingly. We have confirmed that each link resolves properly in a standard web browser.<br>We have also corrected the formatting inconsistency in Reference [37], ensuring that the author name now appears with the appropriate capitalisation ("Singh, J." instead of "singh, j.").
**Areas for improvement (minor language and stylistic edits):**<br>- While the manuscript is generally polished, there are a few minor grammatical and stylistic issues that should be corrected at revision for clarity and precision:<br>-- Article usage and syntax:<br>Example: "Coverage must be optimum to realise its life-saving potential" → should read "Coverage		Thank you for drawing attention to these minor language and stylistic issues. We have now undertaken a careful copyedit of the manuscript to ensure consistent and precise use of English throughout. Specifically, we corrected article usage and syntax (e.g., changing "optimum" to "optimal") and rephrased sentences for smoother readability.

must be optimal to realise its life-saving potential."<br><br>Example: "Invalid doses were common, reaching 2.6% (29/1,111) for OPV" → could be rephrased for smoother readability ("Invalid doses were relatively common, accounting for 2.6% of OPV administrations").		
**Consistency in capitalization and terminology:**<br>--Ensure consistent capitalization of vaccine names (e.g., "Penta3 to MR1" vs "penta3 to MR1") and sub-districts.<br>--Harmonise spelling (e.g., "organisation" vs "organization") according to PLOS' preference for US English.	Entire document	This comment has been addressed in a previous comment
**Minor typographical or punctuation issues:**<br>-- A few misplaced commas and extra spaces (e.g., "immunisation coverage, dropout rate and missed opportunities for vaccination among children aged 12-23 months in the Tamale Metropolis" — add commas for readability).<br>-- Occasional missing spaces before citations (e.g., "[12]and further to 73%").<br><br>- Sentence tightening for conciseness:<br>--Some sentences are overly long or repetitive in the Discussion section; these could be broken into shorter, more direct statements for improved readability.		This has also been addressed in a previous comment<br><br>This has been addressed in a previous comment as well

Title: Consider capitalising consistently (e.g., Timeliness rather than timeliness).	Title and Abstract	This has been addressed in a previous comment
Abstract, lines 30–36: "Coverage must be optimum to realise its life-saving potential" → "Coverage must be optimal to realise its life-saving potential."		This has been addressed in an earlier comment.
Abstract, Results: Add a short quantitative summary of overall fully vaccinated percentage for clarity (e.g., "Only 27.7% of children were fully vaccinated").		A quantitative summary of the overall fully vaccinated percentage has now been added to the results section of the abstract.
Abstract, Conclusion: The phrase "pose significant challenges in the study setting" could be streamlined: "pose major challenges to achieving full immunisation coverage."		Thank you for this helpful suggestion. We have revised the abstract conclusion to streamline the phrasing and ensure clearer alignment with the reviewer's recommendation. Specifically, we replaced the phrase "pose significant challenges in the study setting" with a more direct formulation that emphasises the implications for achieving full immunisation coverage. The revised conclusion now reads:<br>"Persistent gaps in timeliness, high dropout rates, and missed opportunities for vaccination threaten Ghana's ability to achieve IA2030 targets. Strengthening digital immunization registries, defaulter-tracing systems, targeted interventions, and enhanced provider engagement is essential to ensure comprehensive vaccine coverage."
Line 69: "Routine childhood immunisations have significantly reduced morbidity and mortality associated with vaccine-preventable	Introduction	A 2024 WHO fact sheet on immunization coverage has been referenced as recommended

diseases (VPDs) globally.” → You could add a recent 2024 WHO/UNICEF global estimate to strengthen this opening claim.		
Lines 74–80: Long sentence beginning “While LMICs are currently working…” could be split for clarity.		The sentence has not only been split, but it has also been revised to provide clarity as recommended
Line 88: “Conflicts, fragility and inadequate funding” → consider serial comma: “conflicts, fragility, and inadequate funding.”		Thank you for the suggestion. The sentence was revised for clarity, and based on that revision, adding a serial comma does not seem appropriate.
Line 95: “Effective strategies have to be devised, based on current rates of vaccine uptake…” → could be simplified: “Effective strategies must be informed by up-to-date evidence on vaccine uptake.”		The sentence has been revised as follows: “To reverse declining trends and improve immunization coverage in Ghana, effective strategies must be grounded in up-to-date, high-quality and context-specific evidence on vaccine uptake……. ”
Lines 111–114: Clarify whether sampling accounted for cluster design effects in analysis (use of survey weights).	Methods	Thank you for the comment. We have clarified in the analysis section that the analysis accounted for the survey design.
Lines 123–124: “A detailed description of the study area, sampling and data collection have been captured in a publication from the same survey [22].” → should be singular: “has been captured.”		This grammatical error has been corrected
Lines 126–146 (Definitions): Excellent clarity — but please ensure definitions follow WHO phrasing exactly, e.g., “valid dose” and “invalid dose.”		Thank you. The definition section has been revised thoroughly for clarity. To provide additional clarity, a section on reporting recommendation has been added, with appropriate WHO references.
Lines 149–159: The phrase “Data were analysed With the VCQI tool” — lowercase “with.”		That sentence has been revised. The “with” has been replaced with “using” instead.

Line 177 (Ethics): Good inclusion. Suggest adding the date of ethical approval if available		This has been addressed in a previous comment
Line 195: "Immunisation coverage among children aged 12–23 months…" – consider adding N=1,111 to reinforce sample context.	Results	The sample has been added to the heading to reinforce sample context
Table 2: Excellent detail, but column headings could be reformatted for readability (e.g., "Valid Coverage (%)" and "95% CI"). Ensure consistent decimal precision (one decimal place		Column heading has been formatted as recommended for readability. They now read as crude coverage (%) and 95% CI and valid coverage (%) and 95% CI
Lines 214–219 (Timeliness): Sentence "Vittin recorded the highest proportion of invalid doses…" could be simplified for flow: "The Vittin sub-district recorded the highest proportion of invalid doses (5.1%), while Bilpeila recorded the lowest (0.9%)."		We reanalyzed the data and our interpretation of the results are now not presented in this way. As such this does not apply. However, we have endeavoured to ensure that our flow and sentencing is clear and simple.
Fig. 1: Ensure consistent capitalization of vaccine series names (Penta1–3, MR1).		Thank you for the suggestion. Vaccine series names have now been capitalized as recommended.
	Discussion	
Lines 263–266: "Crude coverage rates… were high. Though slightly below national and regional targets…" → Combine into one smoother sentence: "Crude coverage rates were high but remained slightly below national and regional targets of 95%."		We have revised the discussion section, incorporating all the helpful suggestions. That part of the discussion now reads: "Crude coverage rates for vaccines administered at 6, 10, and 14 weeks were relatively high but remained slightly below national and regional targets (coverage of at least 95%) and estimates of the 2022 Ghana Demographic and Health Survey."
Lines 283–289 (Timeliness): "A notable proportion of the children… received invalid doses." → Add a		Thank you for this helpful suggestion. We have revised the manuscript to include an additional explanatory

short comment linking to potential service delivery issues.		sentence linking the occurrence of invalid doses to potential service delivery quality gaps. Specifically, we now highlight that invalid doses may reflect challenges such as inadequate adherence to the national immunization schedule, limited provider training on minimum age and interval requirements, workload-related errors during busy immunisation sessions, or weaknesses in supervision and quality assurance processes
Lines 300–312: The discussion of dropout determinants could cite Ghana-specific socio-demographic studies or GHS reports beyond [36] to enrich interpretation.		Thank you for the suggested references. Four additional Ghana-specific studies have been cited to support the interpretation.
Line 324: Typo: "where children where children were eligible" → remove repetition ("where children were eligible").		The repetition has been removed
Lines 335–338: "Health workers must be equipped…" – this paragraph is strong; consider tightening the last sentence for impact: "Reliable vaccine supply chains and supportive supervision are essential to prevent stockouts and ensure timely vaccination."		The entire discussion section on missed opportunities for vaccination has been revised to enhance clarity and context. As a result, the suggested revision no longer applies.
Lines 341–354 (Strengths & limitations): Well written; you could add one line acknowledging that the exclusion of birth doses and reliance on card data may underestimate true coverage.		Thank you for this valuable suggestion. We have revised the *Strengths and Limitations* section to acknowledge that excluding birth doses and relying primarily on child health card data may underestimate true immunization coverage
Lines 356–365: The conclusion could end more assertively by	Conclusion	Thank you for this helpful suggestion. We agree that reinforcing the policy

linking to policy or IA2030 targets, e.g., "Strengthening tracking systems and community engagement will be essential to achieve Ghana's IA2030 coverage targets."		relevance of our findings and aligning the conclusion with IA2030 priorities will strengthen the manuscript. We have therefore revised the final paragraph of the Conclusion to end with a more assertive, forward-looking statement.
**Reviewer #3**		
The current Data Availability Statement ("Data... can be obtained from the corresponding author upon reasonable request") is not compliant with PLOS policy. The authors must deposit the minimal, anonymized dataset required to replicate the study's findings in a public repository (e.g., Dryad, Zenodo, Figshare) and update the Data Availability Statement in the manuscript to provide the repository name and persistent DOI/accession number;		This has been addressed in an earlier comment.
As this is a cross-sectional observational study, it should be reported in accordance with the STROBE guidelines.<br>Please add a sentence to the Methods section confirming adherence to STROBE and please also provide a completed STROBE checklist as a supplementary information file.		A sentence on the use of the STROBE guidelines has been added, cited, and the checklist has been attached.
The authors state a "validated questionnaire" was used. To ensure full reproducibility, which is a core tenet of PLOS, please provide this questionnaire as a supplementary information file.		The questionnaire has been uploaded as a supplementary information file

Minor issues: 1. Definition of fully vaccinated is fantastic but results in a low 16.0% coverage that might be miscompared to the traditional (and less stringent) global benchmark (e.g., DTP3/MCV1). Please add a sentence in the Methods or Discussion to briefly contextualize this, noting that your definition is based on the full national schedule and is therefore more comprehensive than many standard global reports, therefore not comparable		Thank you for this helpful suggestion. In the methods section, under indicator definitions, we have amended the definition of fully vaccinated as recommended. First, we provided a brief background of full vaccination and the existing variations since the inception of the EPI program and transitioned to clearly describe which doses were included for fully vaccinated children in the study setting. We emphasized that, although this study provides a more comprehensive estimate of fully vaccinated children, it cannot be compared with many global reports.
2. Clarify denominators in table 2. In Table 2, the denominators for coverage correctly decrease for later vaccines (e.g., n=790 for Penta1, n=207 for MR2). This is because not all children in the 12–23-month cohort were old enough for the 18-month vaccines. Please add one sentence in the "Statistical analysis" or Results section (before Table 2) to explain this for the reader (e.g., "Denominators vary by vaccine, as coverage was calculated based on the number of children old enough to be eligible for that specific dose at the time of the survey.		Thank you for the suggested revision. We have added a sentence in the statistical analysis section to indicate that the denominator for the indicator estimates vary by vaccine because coverage was calculated based on the number of children old enough to be eligible for that specific dose at the time of the survey.
3. Our results show a striking drop-off for Rota3 (43.6% coverage) and a massive 52.9% MoV rate. This is a much poorer performance than Penta3 (81.9%) or PCV3 (74.0%), which are given at the same visit. This is a critical quality-of-care		Thank you for the suggestion. We have revised the discussion to point out the need for further investigation to unravel the underlying causes of the discrepancy between the third dose of the rotavirus vaccine and other third doses administered at the same time.

finding. Please add 1-2 sentences to the Discussion pointing out about the need to further explore the root-causes on this specific discrepancy (e.g., Rota-specific stockouts, confusion over oral vs. injectable administration, etc.).		
5. The methods refer to a previous publication "[22]" for a detailed sampling description. While this is fine, the manuscript would be rigorous if you add 2-3 sentences to the "Study area, sampling and data collection" section summarizing the multi-stage sampling process. Typos and formatting should also be fixed on line 344, Table 4 and Figure 1		Thank you. Additional information on the multi-stage sampling technique has now been provided in that section.
**Reviewer #4**	Introduction	
The authors provide a good overview of immunisation coverage and the need for updated evidence. While contextual Ghanaian emphasis is well laid out, authors do not provide information on why this research matters for the Tamale Metropolis. This is important because reports from Ghana's DHS already provide information on immunisation trends, from which dropouts and missed opportunities can be inferred. If this research focuses on Tamale, why is this the case? Are there peculiar coverage issues in Ghana's Northern region that warrant this additional research and new data collection? Is there consistently low vaccination uptake in the sub-context? Are current nationally representative data		Thank you for this thoughtful and important observation. We agree that clearly articulating the rationale for focusing on the Tamale Metropolis strengthens the relevance and contribution of the study. In response, we have expanded the Introduction and Rationale sections to explain why this sub-context warrants specific analysis beyond what is available from national surveys such as the Ghana Demographic and Health Survey (DHS). We have highlighted the need to understand subdistrict and CHPS level immunisation performance and the need for up-to-date, high-quality and context-specific evidence to guide programming and decision making as the rationale for undertaking this study in the Tamale Metropolis

sources inadequate? Is there a gradual increase in certain childhood illnesses that warrant advocacy for better immunisation efforts in the metropolis?		
Lines 101-103: It is unclear what authors mean by "household surveys are limited, particularly in northern Ghana, where disparities in vaccine uptake persist". Ghana's demographic and health surveys (DHS) as well as multiple indicator cluster surveys (MICS) are household surveys which cover the Northern region as well. The statement is therefore not substantive. Is there literature to substantiate the claim? Furthermore, it is important to provide additional information on why a new community-based cross-sectional survey is warranted when information can be derived from the DHS/MICS. Are there key peculiarities for the Tamale Metropolis that needs to be highlighted?		Thank you for the helpful comment. Our intention was not to imply that DHS/MICS exclude the Northern Region, but rather to note their programmatic limitations for metro- and sub-district decision-making. We have revised the text to explicitly state these points rather than the shorthand "household surveys are limited."
Lines 76 and 79: Do you mean missed "opportunities" rather than "communities"?		Thank you for the note. We meant "missed communities." It is a common term that is used to refer to communities that are not in contact with immunization services, particularly hard-to-reach communities.
Lines 89: Can you provide the full meaning of VPDs since they appear for the first time in the work?		The full meaning of VPDs has been defined in the introductory section.
First, the section labelled "study design" is misleading, given that it captures information mainly on	Materials and methods	The study design has been revised to study design and population. That section now reads: "We conducted a

sample size. Even for this, there is no substantive explanation for the sample used for this research. How did the authors determine the sample size of 1602 for their survey? What contributed to the final subsample of 1111 children? The attached data has rows up to 1534. Why is this the case?		descriptive cross-sectional study of 1,020 children aged 12-59 months to enable us to estimate full immunization coverage, describe immunization patterns, and pinpoint operational bottlenecks in routine childhood immunization in the Tamale Metropolis from November to December 2022. This study was carved out of a big community-based cross-sectional study involving 1,577 children aged 0-59 months in the Tamale Metropolis. Children less than 12 months were excluded because they had not reached the age at which completion of the routine immunization schedule could be assessed."
Second, little information is given on how the multi-stage sampling was done. How many stages? How was the sampling frame derived? What approach was used in identifying and selecting participants? Were participants sampled randomly? Additionally, the timeframe for data collection is unclear and somewhat suspicious.		This has been addressed in an earlier comment
The authors report that they collected the data between 21st December 2022 and 10th January 2023; however, the attached data (Excel) shows dates before this reported period (29th November - 12th December). Is there a reason for this?		Thank you. We have revised the manuscript, including the study design and ethical consideration sections to include the correct dates of data collection.
The authors also report that the cross-sectional cluster survey consisted of 1602 children aged 0-59 years but it is concerning that out of this sample, 1111 were children		Thank you for the vital feedback. We checked the dataset, reran the analysis and now included children aged 12-59 months in the study population, because we intended to estimate full

aged 12-23 months (about 70% of the whole sample). Was the sampling approach not random? There is also no justification for focusing on those aged 12-23 months.		immunization coverage. The estimated sample and the enrolled sample descriptions have been described in both the statistical analysis section and the results section. The overall sample description has now been captured in the results section. The justification for including children aged 12-59 months has been provided.
Third, the terms need to be well-defined, and authors should provide references to support them. For instance, missed opportunities are defined as children not receiving all age-appropriate vaccines per the immunisation schedule. Do these include drop-outs from multi-dose vaccines or when the child misses out on all the doses of a vaccine entirely? Or does this include missing the age-appropriate timing for the vaccine? Do authors capture children who are aged 12-17 months but have not yet taken MR2 (taken at 18 months) as missed opportunities? This is not well defined.		Thank you. References have been provided for indicator definitions. Further details have also been provided for the indicator definitions for clarity.
Additionally, how do authors deal with this (children aged 12-17 months who have not taken MR2 yet) when calculating "full vaccination"? Are they automatically captured as not fully vaccinated?		Thank you for raising this important clarification. We have now specified our approach more clearly in the Methods and Definitions sections.
For invalid doses, the term only captures instances where the dose is given before the right age or when its <28days in between doses. Why are those given after the scheduled dates/time not included?		We appreciate the opportunity to clarify this point. Our definition of invalid doses follows WHO and Ghana EPI guidance, which consider a dose “invalid” when it fails to meet the minimum age or minimum interval

		requirements. Late doses, often called delayed doses, were categorized under timeliness, not invalidity, because they remain valid per WHO immunological criteria.
How did authors validate data on invalid doses?		Thank you for this important query. We have expanded the methods section, particularly the statistical analysis section, to clarify the procedures used to validate dose-timing information and ensure accurate identification of invalid doses. We clearly indicated that we used the vaccination dates provided on the health records of the children to validate invalid doses.
This particular term (invalid doses) is used consistently to infer timeliness of vaccine uptake in the results section, however, the concept of vaccine timeliness extends beyond what "invalid doses (as currently defined)" capture.		We appreciate the reviewer's point. The manuscript has been revised to ensure that invalid doses are not used as a proxy for overall timeliness, but rather as a specific, well-defined indicator of early administration errors within the broader timeliness framework.
Fourth, the authors do not provide adequate information on how the data was weighted. Why were subsets not weighted?		This has been addressed in an earlier comment
The authors also indicate that they did not include vaccines taken at birth. However, these vaccines are included in internationally defined vaccination markers. Why were those vaccines (e.g. BCG) excluded? To what extent does the omission of BCG vaccines and OPV0 vaccines affect vaccine coverage estimates?		We have now included birth doses in the computation of vaccine-specific coverage indicators.
Lastly, the ethical considerations need further clarification. Authors		Thank you for the helpful comment. We have provided clarification in the ethical

need to provide additional information on what they mean by the rights of respondents. How was voluntary participation, anonymity and confidentiality ensured? The data attached has information on respondents' telephone numbers and house addresses, which is not appropriate given the importance of anonymity in research. Did participants also consent to having their personal details publicly available? How was data managed and protected?		consideration section on all the queries raised. We made also now removed all identifiers in the dataset.
Lines 185-186: The interpretation does not come out clearly. Do you mean that 99% of the mothers attended antenatal care? Authors could take time to break long sentences into simple sentences for better clarity.	Results	Thank you for this helpful observation. We agree that the original sentence was unclear and overly long. We have now revised it to improve clarity and readability.
The results in Table 2, specifically those for "fully vaccinated" and "not fully vaccinated," require revision. Authors stated in the methods section that “not fully vaccinated” is the opposite of being “fully vaccinated”, which has a binary inference unless otherwise. The estimates for "not fully vaccinated" do not match up then. If about 27.7% of children have been fully vaccinated, then 72% have not been fully vaccinated. However, the percentages reported do not support this observation		This has been addressed in an earlier comment
Does the authors’ definition of “not fully vaccinated” mean zero doses		This has also been addressed in an earlier comment

(if this is the case, the estimates do not measure up either when more than 80% of children have taken at least one vaccine)? Authors need to provide more precise definitions and re-analyse the work to ensure appropriate estimates.		
Furthermore, in Table 2, the weighted sample size is 111 for both crude coverage and valid coverage. Is this a mistake?		Thank you for this important observation. We appreciate the opportunity to clarify the presentation of sample sizes in Table 2. We have revised this section thoroughly and the sample size is now 1,020. However, the weighted sample for both crude and weighted remains the same as we report coverages from card only, meaning that it is the same analytical population. This explanation has been provided in the methods section.
The concept of vaccine timeliness used in this research does not align with international usage of the term and may require the use of an alternative term that fits the study – “invalid doses”. I observe that the percentages reported as invalid doses in Table 3 do not substantiate the estimates reported as "Valid coverage" in Table 2, especially when valid coverage is calculated as “Crude coverage – invalid doses”. For example, the average 2.5% observations reported as invalid for Penta vaccines in Table 3 seem under-estimated. From Table 2, Penta 3 crude coverage is 81.9% and the valid coverage is 63.8%.		This has been addressed in an earlier comment.
Given how authors define valid coverage, this means that invalid dose for Penta 3 alone (difference		This has been addressed in an earlier comment

between coverage and valid coverage) will be about 18%. This estimate is higher than what authors report as the average invalid dose for Penta vaccines. It could be that authors are not providing better explanation for their estimates.		
Authors are encouraged to provide additional supplementary information to explain how they arrived at the estimates		Thank you for the helpful feedback. Additional information has been provided on how we generated the estimates.
Drop-out rates: Are drop-out rates based on crude or valid coverage? Authors seem to provide overreported estimates. For instance, the descriptive estimates from Table 2 show that uptake of OPV1 is 91.3% and OPV3 is 72.1% (based on crude coverage). This means that the drop-out rate is ~19.2% but the authors report 22.3% (the estimates do not tally either when valid coverage is used). The same issue is observed for the other multi-dose vaccines. It is also unclear why authors would want to draw estimates between Penta 3 and MR1 as these are entirely different vaccines. Furthermore, there is no information for other multi-dose vaccines such as Rota and PCV. Why is this the case?		Thank you. Drop-out estimates were based on crude coverages, and this clarification has been provided in the methods section. We reanalysed the dataset and these estimates have changed.
Missed opportunities: Authors could report percentages of actual missed opportunities. If the missed opportunities were corrected (which is unclear what this means), then they are no longer missed opportunities.		We appreciate the reviewer's comment and have revised the terminology, clarified definitions, and added percentages and number of actual MOVs. The revised presentation now clearly distinguishes between missed

		opportunities and subsequent correction, in line with WHO standards.
Since the authors collected information on some demographic characteristics, why was this not captured in the analysis?		Thank you for this observation. Demographic data on the survey sample has been provided in the results section.
The discussion is good; however, it could be strengthened further. The authors could explain why reporting crude and valid vaccination coverage matters and recommend which is more appropriate. Given the issues raised in the methods and results section, the authors may need to revisit the discussion write-up.	Discussion	We appreciate the reviewer's suggestion. The Discussion has been revised to clearly articulate the distinctions and relevance of crude versus valid coverage and to recommend the appropriate use of each measure.
**Reviewer # 5**		
Title: Immunisation Coverage, Dropout Rate, Timeliness and Missed Opportunities for Vaccination Among Children Aged 12–23 Months in Tamale Metropolis, Ghana<br><br>Suggested title: Routine Childhood Immunisation Coverage, Dropout, and Missed Opportunities in Northern Ghana: Evidence from a Community-Based Survey in Tamale Metropolis.		The suggested title has been adopted
Study Rationale and Framing: The introduction appropriately identifies declining vaccination coverage in Ghana but could more clearly articulate why Tamale Metropolis warrants focused investigation beyond general low coverage in northern Ghana. Adding context on sub-regional inequities or service-		Thank you. This has been addressed in a previous comment.

delivery characteristics would justify the site selection.<br>The objectives are stated clearly, but the novelty could be enhanced by framing the study as part of broader post-COVID-19 immunisation recovery or IA2030 monitoring.		
The authors mentioned that "multi-stage sampling" was used. This is not enough and should be detailed. What were the stages of sampling they followed, and how did they control against the possibility of introducing biases?	Methods	Thank you. This has been addressed in a previous comment.
The use of WHO's VCQI tool in Stata 15.1 is appropriate and well-documented. However, the denominator definition and handling of missing vaccination records require brief clarification (e.g., whether "not seen" cards were excluded or coded as missing).		Thank you. We have now clarified our denominator definition and how we handled missing or unseen vaccination records in the statistical analysis section.
The description of timeliness and invalid doses is clear, but the justification for the <28-day threshold should explicitly cite the WHO reference manual (2018). The authors note that birth doses were excluded; this should be acknowledged as a limitation in interpreting "fully vaccinated" status.		Thank you. This has been addressed in a previous comment
Please indicate how you handled missingness/missing data. Although you referenced the VCQI tool, none of your tables include information on missing data. If you excluded vaccination data from birth and captured only from 12 to 23 months,		Thank you. This has been addressed in a previous comment.

that cannot compensate for handling missing data that may have occurred within the age range you captured.		
The Tables are clear and consistent with WHO coverage reporting standards. However:<br>Please add 95% confidence intervals for fully vaccinated/not fully vaccinated in Table 2 to show uncertainty.	Results	Thank you. The 95% confidence intervals for the fully vaccinated and not fully vaccinated has been added.
Figure 1 should include absolute denominators (n/N) for transparency.		Thank you. The analysis tool produces graphs in this format (%, N). The control program for the analysis, particularly on the suggested revision, cannot be modified by users.
The authors' reporting of invalid doses and missed opportunities is valuable, but MoV denominators (number of eligible visits) should be clearly defined for reproducibility.		Thank you. We agree that explicit definition of MOV denominators is essential for reproducibility and have now clarified this in the methods, particularly the statistical analysis section.
**Discussion and Interpretation**: The discussion correctly identifies systemic factors affecting coverage (provider practices, caregiver engagement, logistics). However:<br>Several interpretations (e.g., "female children have lower dropout risk") cite general Ghanaian evidence that may not derive from the study's dataset—this should be qualified as secondary literature, not inference. The discussion could benefit from connecting results to specific IA2030 strategic priorities (equity, life-course vaccination, data use). The implications section would be stronger if it linked the findings to concrete local actions	Discussion	Thank you for pointing this out. We have rephrased those sections to explicitly state that these observations are drawn from secondary literature, not from our results. We have also aligned our discussion with IA2030 priorities.

(e.g., the use of digital registries, outreach campaigns, and community health volunteers).		
Limitations: The limitations section is transparent but can be condensed. Emphasize that reliance on home-based records minimizes recall bias yet limits representativeness for cardless children, potentially underestimating missed opportunities.		Thank you. This has been addressed in a previous comment.
**Minor Comments:**<br>- Define abbreviations upon their first use, and avoid abbreviations in the Abstract, e.g., MoV, MR1		Thank you. This has been addressed in a previous comment.
State the IA2030 goals/targets in lines 90-91, so that readers can have a better idea of how they compare with the current rates in the population. It is not enough to only cite the document.		Thank you. This has been addressed in a previous comment.
Ensure consistent use of capitalization in vaccine names (e.g., "Penta3," "OPV3," "Rota3").<br>- Correct minor typographical errors (e.g., repeated "where children where children".		Thank you. This has been addressed in an earlier comment
The term "not fully vaccinated" might be better described as "partially vaccinated" for readability.		Thank you. This has been addressed in a previous comment.
- Verify numeric consistency: weighted sample sizes in Table 2		Thank you. This has been addressed in a previous comment.

appear truncated. Please clarify whether it is “111” or “1,111”.		
The reference list is thorough and current (up to 2025), but please ensure journal titles follow PLOS formatting (italicized, full names).		Thank you. The PLOS recommended referencing style was used for inserting references with a reference manager.
Ethical and Data Transparency: The manuscript states approval from the Navrongo Health Research Centre IRB (NHRCIRB495) and that data are available upon request. Although this is adequate per PLOS Global Public Health standards, the authors should consider making anonymized data openly available via an institutional repository to align with PLOS’s “minimal dataset” policy.		This has been addressed in an earlier comment
